# Moving forward together: collaborative strategies in HIV prevention across Africa – a narrative review

**DOI:** 10.1097/MS9.0000000000002961

**Published:** 2025-01-31

**Authors:** Emmanuel Ifeanyi Obeagu, Getrude Uzoma Obeagu

**Affiliations:** a Department of Biomedical and Laboratory Science, Africa University, Mutare, Zimbabwe; b School of Nursing Science, Kampala International University, Ishaka, Uganda

**Keywords:** Africa, collaboration, community engagement, HIV prevention, public health

## Abstract

HIV remains a major public health challenge across Africa, with the region accounting for nearly 70% of the global HIV burden. Despite significant advances in HIV prevention, treatment, and care, the epidemic continues to disproportionately affect certain populations, exacerbating health disparities and posing a barrier to socioeconomic development. This narrative review explores collaborative strategies in HIV prevention across Africa, focusing on multi-sectoral approaches, community-based interventions, and international partnerships. The review emphasizes the critical role of local communities, healthcare systems, and government organizations in reducing HIV transmission, with a particular focus on the importance of tailored prevention programs for vulnerable populations. Key strategies highlighted in the review include the integration of HIV prevention into broader public health initiatives, such as reproductive health, education, and poverty reduction programs. Community-based interventions, especially those involving community health workers (CHWs) and peer education, have proven effective in reaching at-risk populations. Additionally, international partnerships and funding from organizations like PEPFAR and the Global Fund have been instrumental in scaling up HIV prevention efforts, providing essential resources and technical support. Regional collaborations, such as those between neighboring countries in East and Southern Africa, have also played a vital role in addressing cross-border transmission and providing services to migrant populations.

## Introduction

HIV/AIDS remains one of the most significant public health challenges across Africa, where the burden of the epidemic is particularly acute^[1]^. Despite decades of concerted efforts, the continent continues to experience high rates of HIV prevalence and new infections. This enduring crisis necessitates innovative and collaborative strategies to curb the spread of HIV and improve the quality of life for those living with the virus^[2]^. As the global health community rallies to support Africa in this fight, the emphasis has increasingly shifted toward multi-sectoral partnerships and community-driven initiatives. Collaboration has proven to be a cornerstone in the battle against HIV/AIDS^[3]^. By combining the resources, expertise, and influence of various stakeholders – including governments, non-governmental organizations (NGOs), international agencies, community groups, and the private sector – more comprehensive and sustainable interventions can be developed. These collaborative strategies have facilitated a more effective and cohesive response to the epidemic, addressing not only the medical aspects but also the social, economic, and cultural dimensions of HIV prevention. Community engagement and education are pivotal components of these collaborative strategies. Local communities possess unique insights into the cultural and social dynamics that influence behavior and attitudes toward HIV^[4]^. Engaging communities in the design and implementation of prevention programs ensures that interventions are culturally sensitive and more likely to be accepted. Grassroot organizations and community health workers are at the forefront of these efforts, playing a crucial role in raising awareness, reducing stigma, and promoting safer sexual practices. Educational initiatives have been particularly effective in reducing the transmission of HIV^[5]^. Programs that provide accurate information about HIV transmission, prevention methods, and the importance of regular testing have empowered individuals to take proactive steps in protecting their health. For instance, the “ABC” approach (abstain, be faithful, and condomize) used in Uganda has significantly contributed to lowering the HIV prevalence rate. Peer education programs, especially among youth, have also been successful in fostering a supportive environment where open discussions about HIV are encouraged.

**HIGHLIGHT**

- **High Prevalence**: Sub-Saharan Africa bears 70% of global HIV burden.
- **Key Populations**: Women, adolescents, MSM, sex workers, and PWID are disproportionately affected.
- **Effective Interventions**: ART expansion, PMTCT, VMMC, and PrEP show a positive impact.
- **Challenges**: Stigma, healthcare infrastructure, funding sustainability, and political instability persist.
- **Future Directions**: Enhanced community engagement, innovative financing, and integrated healthcare are crucial for sustainable HIV/AIDS response in Africa.

Integration of HIV prevention and treatment services represents another key strategy in the collaborative response to HIV in Africa^[6]^. The “test and treat” approach, which advocates for immediate treatment following a positive HIV diagnosis, has been widely endorsed and implemented. This strategy not only helps in managing the health of those infected but also reduces the likelihood of further transmission. Countries like Botswana have seen marked improvements in HIV-related health outcomes due to the successful implementation of this integrated approach. Decentralized health services, such as mobile clinics, have further enhanced the reach and accessibility of HIV prevention and treatment services^[7]^. By bringing these services closer to remote and underserved populations, geographical barriers are reduced, and more individuals can benefit from timely interventions. The role of governments and NGOs in establishing and supporting these mobile and community-based health services cannot be overstated, as they provide crucial infrastructure and resources. Policy and advocacy efforts have also been instrumental in shaping the HIV prevention landscape in Africa^[8]^. Comprehensive national HIV/AIDS strategies, developed with the support of international organizations like UNAIDS and the Global Fund, provide a roadmap for effective intervention. These policies prioritize prevention, care, and treatment, ensuring that resources are allocated strategically to address the epidemic comprehensively. Advocacy groups, often comprising civil society organizations, have been pivotal in influencing policy changes and securing funding, ensuring that HIV remains a priority on national and international agendas. Public-private partnerships (PPPs) have emerged as a powerful mechanism in advancing HIV prevention^[9]^.

## Epidemiological empirical review of HIV in Africa

The human immunodeficiency virus (HIV) epidemic remains one of the most significant public health challenges in Africa^[10]^. Despite substantial efforts in controlling the spread of the virus and improving the health outcomes of those affected, the continent continues to bear a disproportionate burden of the global HIV/AIDS epidemic. This review provides an empirical examination of the epidemiology of HIV in Africa, highlighting the prevalence, incidence, key populations at risk, and the effectiveness of various intervention strategies. HIV prevalence and incidence rates in Africa vary significantly across regions and countries. Sub-Saharan Africa is the most affected region, home to approximately 70% of the global HIV-positive population^[1]^. According to UNAIDS data, as of 2023, there are an estimated 25.7 million people living with HIV in sub-Saharan Africa, with East and Southern Africa being the most affected subregions. In East and Southern Africa, countries like South Africa, Mozambique, and Zambia report some of the highest prevalence rates, with adult prevalence rates exceeding 15% in some areas^[10]^. West and Central Africa, while also heavily impacted, generally have lower prevalence rates, with countries such as Nigeria and Cameroon experiencing significant, but relatively lower, epidemic burdens^[11]^. The incidence of new HIV infections has seen a notable decline over the past two decades due to intensified prevention efforts. However, the rate of new infections remains high, with approximately 1.1 million new infections reported in 2022^[12]^. Southern Africa continues to record the highest number of new infections annually, underscoring the need for sustained and enhanced prevention strategies. In sub-Saharan Africa, women and girls account for nearly 60% of all new HIV infections. Young women (age 15–24) are particularly vulnerable due to a combination of biological, social, and economic factors^[13]^.

Men who have sex with men (MSM) face a significantly higher risk of HIV infection compared to the general population, exacerbated by stigma, discrimination, and legal barriers that limit access to prevention and treatment services^[14]^. Sex workers and their clients contribute significantly to the epidemic. Stigma and criminalization of sex work hinder access to healthcare services for this group. Although less prevalent in Africa than in other regions, people who inject drugs (PWID) are at heightened risk due to shared needle use and limited harm reduction services. This group remains at high risk due to inadequate access to sexual health education and services, as well as socio-economic vulnerabilities. The expansion of antiretroviral therapy (ART) has been a cornerstone of the response to HIV in Africa^[15]^. Countries like Botswana and Rwanda have achieved near-universal ART coverage, leading to significant declines in HIV-related morbidity and mortality. However, challenges such as drug resistance, treatment adherence, and sustainable financing persist. Prevention of mother-to-child transmission (PMTCT) programs have markedly reduced new HIV infections among infants. In countries like South Africa, the implementation of comprehensive PMTCT programs has led to transmission rates of less than 5%^[16–19]^.

Voluntary medical male circumcision (VMMC) has been shown to reduce the risk of heterosexual HIV transmission^[20]^. Programs in countries like Kenya and Uganda have scaled up VMMC services, contributing to declines in new infections. Pre-exposure prophylaxis (PrEP) has emerged as an effective prevention strategy for high-risk populations. Programs in countries such as Kenya and South Africa are scaling up PrEP delivery, although uptake and adherence challenges remain^[21]^. Educational campaigns and community-based interventions aim to change behaviors that increase HIV risk^[21]^. These include promoting condom use, reducing multiple sexual partnerships, and increasing HIV testing and counseling. While these interventions have had some success, their impact varies based on cultural and social contexts. Efforts to implement harm reduction strategies such as needle exchange programs and opioid substitution therapy have been limited but are gaining traction in some regions. Greater investment and policy support are needed to expand these services. The epidemiology of HIV in Africa underscores the complexity and magnitude of the epidemic. While significant strides have been made in reducing prevalence and incidence rates and expanding access to treatment, substantial challenges remain. Addressing these challenges requires a continued, multifaceted approach that includes strengthening healthcare systems, combating stigma, ensuring sustainable funding, and fostering political commitment. By leveraging the successes and addressing the ongoing barriers, Africa can continue to make progress toward controlling and ultimately ending the HIV/AIDS epidemic.

## Aim

The aim of this epidemiological empirical review is to comprehensively analyze the current state of HIV in Africa, focusing on prevalence, incidence, key populations at risk, intervention strategies, challenges, and future directions.

## Review methodology

### Search strategy

1. **Database Selection:** A systematic search was conducted using academic databases such as PubMed, Google Scholar, Scopus, and WHO Global Health Library. These databases were chosen for their comprehensive coverage of peer-reviewed literature, reports, and studies related to HIV/AIDS in Africa.
2. **Search Terms:** The search strategy included a combination of Medical Subject Headings (MeSH) terms and keywords related to HIV/AIDS (e.g. “HIV,” “AIDS,” and “HIV infections”) and Africa (e.g. “Africa,” “sub-Saharan Africa,” and “West Africa”). Boolean operators (AND, OR) were used to refine search results and ensure relevance.
3. **Inclusion Criteria:** Studies and reports included in the review met the following criteria:

- Focus on HIV/AIDS epidemiology in African countries or regions.
- Published in peer-reviewed journals, official reports, or reputable organizations.
- Available in English or with English translations.

1. **Exclusion Criteria:** Studies were excluded if they did not focus specifically on HIV/AIDS epidemiology in Africa or if they were not available in English or did not have English translations.

**Quality Assessment**: The quality of included studies was assessed based on study design, methodology, sample size, and potential biases. Studies were critically appraised to ensure reliability and validity of the findings.

## Community engagement and education

Community engagement and education are fundamental pillars in the collaborative efforts to prevent HIV across Africa^[22]^. These strategies leverage the unique strengths and insights of local communities, making interventions more culturally relevant and effective. Engaging communities directly in the fight against HIV ensures that prevention programs are not only accepted but actively supported by those they aim to help. One of the key benefits of community engagement is the ability to tailor education initiatives to the specific social and cultural contexts of different regions. Local leaders, grassroot organizations, and community health workers are instrumental in this process. They understand the local norms, beliefs, and practices that influence behavior, and they can communicate HIV prevention messages in ways that resonate with their communities. For instance, in rural areas of Kenya, community health workers have successfully used storytelling and local proverbs to convey the importance of HIV testing and safe sexual practices, making the information more relatable and impactful. Educational programs within communities play a crucial role in raising awareness about HIV transmission and prevention methods^[23]^. These programs often include workshops, seminars, and informational campaigns that provide accurate and up-to-date information. The “ABC” (abstain, be faithful, and condomize) approach used in Uganda is a notable example. This strategy has been credited with significantly reducing the HIV prevalence rate by promoting abstinence, fidelity, and condom use through widespread community education efforts.

Peer education programs are particularly effective among younger populations. These programs train young people to become educators and advocates for HIV prevention within their peer groups^[24]^. In South Africa, peer educators have played a vital role in reducing stigma and encouraging open discussions about HIV^[25]^. By using social networks and peer influence, these programs create a supportive environment where young people feel comfortable seeking information and services related to HIV prevention. This approach not only spreads knowledge but also builds a community of support that reinforces positive behaviors. Community engagement also helps in reducing the stigma and discrimination associated with HIV. Stigma remains one of the biggest barriers to effective HIV prevention and treatment^[26]^. By involving community members in education and awareness campaigns, these initiatives humanize the issue and challenge misconceptions about the virus. In Nigeria, for example, community-based organizations have organized events that include testimonies from people living with HIV, fostering empathy and understanding among the public. These efforts help to break down the barriers that prevent individuals from seeking testing and treatment.

Moreover, community engagement ensures that HIV prevention programs are sustainable and have long-lasting impacts^[30]^. When communities are actively involved in the planning and implementation of these programs, they are more likely to take ownership and continue the efforts even after external support diminishes. This local ownership is critical for the sustainability of HIV prevention initiatives. In Tanzania, community-led health committees have been established to oversee and sustain HIV prevention activities, demonstrating the long-term benefits of community involvement. Partnerships between communities and external organizations, such as NGOs and international agencies, further enhance the effectiveness of education and engagement strategies. These partnerships provide resources, training, and technical support while ensuring that programs remain community-driven^[27]^. The African HIV/AIDS Prevention Network (AHAPN) is an example of such collaboration, where local communities work alongside international partners to develop and implement prevention programs that are culturally appropriate and effective. In addition to traditional methods, innovative approaches such as digital media and mobile technology are being increasingly utilized to engage and educate communities about HIV. Mobile health (mHealth) platforms offer new opportunities for reaching wider audiences with prevention messages and information. In Kenya, the “mHealth Kenya” initiative uses SMS and mobile apps to provide HIV education, reminders for medication adherence, and updates on local testing and treatment services. These digital tools enhance the reach and accessibility of HIV prevention efforts, especially among younger and more tech-savvy populations.

## Integration of HIV prevention and treatment services

The integration of HIV prevention and treatment services is a critical strategy in the fight against the HIV/AIDS epidemic across Africa^[28]^. This approach ensures that individuals not only have access to preventative measures but also receive timely and effective treatment if they test positive for HIV. By bridging the gap between prevention and treatment, integrated services can significantly reduce HIV transmission rates and improve the overall health outcomes for people living with HIV. One of the most effective models of integrated HIV services is the “test and treat” strategy^[29]^. Endorsed by the World Health Organization (WHO), this approach recommends that individuals who test positive for HIV begin antiretroviral therapy (ART) immediately, regardless of their CD4 count. The immediate initiation of ART reduces the viral load in individuals, thereby decreasing the risk of HIV transmission to others. Countries like Botswana have seen significant success with this approach, with substantial reductions in new HIV infections and improvements in the health of those living with HIV.

Mobile clinics and decentralized health services have also played a crucial role in the integration of HIV prevention and treatment^[30]^. These services bring healthcare closer to remote and underserved populations, breaking down geographical barriers that often hinder access to care. Mobile clinics provide a range of services, including HIV testing, counseling, distribution of prevention tools such as condoms, and the provision of ART. In rural areas of Tanzania, for instance, mobile health units have been instrumental in reaching populations that otherwise have limited access to healthcare facilities, ensuring continuous and comprehensive care. Integration efforts extend beyond the healthcare system to include community-based initiatives. Community health workers are trained to deliver both prevention education and treatment services. This dual role not only enhances the reach of healthcare services but also builds trust within the community, as health workers become reliable sources of both prevention and treatment information. In Uganda, community health workers have been key in delivering home-based care, conducting follow-ups for ART adherence, and educating communities about HIV prevention, creating a seamless link between different aspects of HIV care.

Moreover, integrating HIV services with other health services, such as maternal and child health, tuberculosis (TB) treatment, and sexual and reproductive health services, has proven beneficial^[31]^. Co-locating these services reduces the stigma associated with attending HIV-specific clinics and promotes holistic healthcare. For example, in South Africa, integrating HIV services with TB clinics has led to improved detection and treatment of co-infections, which is critical given the high prevalence of TB among people living with HIV. Similarly, integrating HIV testing and treatment with prenatal care ensures that pregnant women receive comprehensive care, reducing the risk of mother-to-child transmission of HIV. The role of technology in facilitating the integration of HIV prevention and treatment services cannot be overstated^[32]^. Digital health platforms, including electronic health records (EHRs) and mobile health (mHealth) applications, have enhanced the coordination of care. These technologies allow for better tracking of patient data, appointment scheduling, and follow-up reminders, ensuring that individuals remain engaged in care. In Kenya, the use of EHRs has streamlined the management of patient information, enabling healthcare providers to deliver more coordinated and efficient care^[33,34]^.

## Policy and advocacy

Policy and advocacy are integral to advancing HIV prevention and treatment across Africa^[3-40]^. These efforts shape the framework within which healthcare services are delivered, ensuring that they are effective, equitable, and sustainable. By influencing policy decisions and advocating for the rights and needs of those affected by HIV, stakeholders can create an environment conducive to comprehensive HIV responses. This section explores how robust policy frameworks and advocacy initiatives have driven progress in HIV prevention and treatment, emphasizing the roles of governments, civil society, and international partners.

## National and regional policy frameworks

National HIV/AIDS strategies, supported by regional and international guidelines, provide the foundation for HIV prevention and treatment efforts^[35]^. These policies outline priorities, allocate resources, and establish targets for reducing new infections and improving the quality of life for people living with HIV. Countries like South Africa and Kenya have developed comprehensive national strategic plans that align with the United Nations’ Sustainable Development Goals (SDGs) and the African Union’s Agenda 2063^[36]^. These plans emphasize the importance of a multi-sectoral approach, integrating HIV services with other health and social services to enhance their reach and impact. Regional collaborations also play a pivotal role in shaping HIV policy. The African Union’s African Strategy on HIV and AIDS 2016–2020, for example, aims to strengthen regional cooperation and harmonize responses to the epidemic^[37]^. By fostering collaboration among member states, these frameworks facilitate the sharing of best practices, resources, and technical expertise. The East African Community (EAC) has similarly developed a regional HIV and AIDS framework that promotes cross-border cooperation, ensuring that interventions are cohesive and effective across different countries.

## Legislative and policy advancements

Advocacy efforts have led to significant legislative and policy advancements that enhance HIV prevention and treatment^[38]^. Key milestones include the removal of discriminatory laws that hinder access to services and the enactment of laws that protect the rights of people living with HIV. In countries like Mozambique, advocacy campaigns have successfully lobbied for the decriminalization of HIV transmission, reducing stigma and encouraging individuals to seek testing and treatment without fear of legal repercussions. Additionally, the introduction of laws mandating comprehensive sexual education and HIV prevention programs in schools has been instrumental in reaching younger populations. Moreover, the inclusion of HIV treatment in national health insurance schemes and the removal of taxes on antiretroviral drugs have been critical in making treatment more affordable and accessible^[39]^. In Kenya, the advocacy efforts of civil society organizations led to the removal of value-added tax (VAT) on HIV medications, significantly reducing the cost of treatment for those in need^[40]^. Such policy changes not only improve healthcare access but also demonstrate a commitment to reducing the economic barriers to HIV care.

## Strengthening health systems and service integration

Effective advocacy has also focused on strengthening health systems and promoting the integration of HIV services with other health and social services^[41]^. This approach ensures that individuals receive holistic care that addresses not only their HIV status but also their overall health and well-being. For example, advocacy by organizations in Uganda has led to the integration of HIV services with maternal and child health programs, enhancing the prevention of mother-to-child transmission of HIV (PMTCT). This integration ensures that pregnant women receive comprehensive care, significantly reducing the risk of transmitting HIV to their children. Furthermore, advocacy efforts have pushed for the inclusion of HIV services in broader health system strengthening initiatives. In Ghana, advocacy by the Ministry of Health and various NGOs has led to the incorporation of HIV services into the country’s primary healthcare strategy, ensuring that HIV care is decentralized and accessible at all levels of the health system^[42]^. This integration is crucial for improving service delivery, particularly in rural and underserved areas.

## Global and regional partnerships

International and regional partnerships have been instrumental in shaping HIV policy and advocacy. Organizations such as UNAIDS, the Global Fund, and PEPFAR have provided substantial financial and technical support to strengthen national HIV responses^[43]^. These partnerships have enabled countries to scale up testing, treatment, and prevention services, leveraging global resources to enhance local efforts. For instance, PEPFAR’s support has been critical in expanding ART coverage in countries like Nigeria and Tanzania, helping to achieve significant reductions in HIV-related mortality and morbidity. Regional bodies, such as the Southern African Development Community (SADC) and the East African Community (EAC), have also played a vital role in advocacy and policy development^50^. These bodies facilitate regional dialogues, share best practices, and coordinate responses to HIV across borders. The SADC HIV and AIDS Strategy, for example, provides a regional framework that promotes coordinated action on key issues such as gender-based violence, which is closely linked to HIV risk.

## Empowering civil society and community voices

Civil society organizations (CSOs) and communities themselves have been at the forefront of HIV advocacy, bringing the voices of those affected by HIV to the policy table^[44]^. These organizations have played a crucial role in raising awareness, lobbying for policy changes, and holding governments accountable for their commitments. In Zimbabwe, advocacy by community-based organizations has led to the establishment of community-led monitoring systems that track the implementation of HIV policies and services, ensuring that they meet the needs of the most vulnerable populations. Additionally, community advocacy has been instrumental in promoting the rights of key populations, including sex workers, men who have sex with men, and people who inject drugs. In Malawi, advocacy efforts by key population groups have led to the development of targeted HIV programs that address the specific needs and challenges faced by these populations, reducing stigma and improving access to services.

## Public-private partnerships

Public-private partnerships (PPPs) have emerged as a vital mechanism in the fight against HIV/AIDS in Africa, combining the resources and expertise of both the public and private sectors to enhance HIV prevention, treatment, and care^[45]^. These collaborations leverage the strengths of various stakeholders, from government agencies and non-governmental organizations (NGOs) to private companies and international organizations, creating a more robust and comprehensive response to the epidemic.

## Enhancing access to HIV prevention tools

One of the primary contributions of PPPs in HIV prevention has been the increased availability and accessibility of prevention tools such as condoms, pre-exposure prophylaxis (PrEP), and educational materials^[46]^. Pharmaceutical companies, in collaboration with governments and NGOs, have played a significant role in making these tools more widely available. For instance, a partnership between the South African government and pharmaceutical companies has facilitated the large-scale distribution of condoms and the implementation of widespread PrEP programs, particularly targeting high-risk populations such as sex workers and men who have sex with men. Moreover, PPPs have enabled the development and dissemination of innovative prevention technologies. Self-testing kits, which allow individuals to test for HIV in the privacy of their own homes, have been made available through partnerships between healthcare companies and NGOs. In Kenya, a partnership with a major healthcare firm has resulted in the distribution of millions of self-testing kits, significantly increasing the reach of HIV testing and early detection efforts.

## Scaling up treatment and care

PPPs have also been instrumental in scaling up access to antiretroviral therapy (ART) and improving the overall care for people living with HIV^[47]^. By pooling resources and expertise, these partnerships have enhanced the supply chain management of HIV medications, ensuring a consistent and reliable supply. In Nigeria, for example, a partnership between the government and a leading pharmaceutical company has streamlined the distribution of ART, reducing stockouts and improving adherence to treatment protocols. These collaborations often extend beyond the provision of medications to include comprehensive care programs that address the broader needs of people living with HIV. For instance, in Ethiopia, a PPP initiative has integrated HIV treatment with nutrition support, mental health services, and economic empowerment programs, providing a holistic approach to care that improves the quality of life for individuals affected by HIV.

## The test and treat approach

The “test and treat” approach, which involves the routine testing of individuals for HIV and the immediate initiation of antiretroviral therapy (ART) for those who test positive, has emerged as a key strategy in HIV prevention and care worldwide. This approach aims to reduce HIV transmission by ensuring that individuals living with HIV are promptly diagnosed and treated, thereby achieving viral suppression and minimizing the likelihood of onward transmission. Globally, the adoption of this approach varies depending on country-level healthcare infrastructure, access to ART, and public health priorities. While the core concept remains consistent, the specifics of treatment regimens and implementation differ across regions, influenced by factors such as available resources, healthcare systems, and patient populations^50^. In high-income countries, the test and treat approach is well-integrated into healthcare systems, often as part of routine screening in clinics and hospitals. ART regimens typically involve a combination of three drugs from different classes, including integrase strand transfer inhibitors (INSTIs), non-nucleoside reverse transcriptase inhibitors (NNRTIs), and nucleoside reverse transcriptase inhibitors (NRTIs). These regimens are highly effective at achieving viral suppression, with INSTIs now considered the preferred first-line option due to their potency, tolerability, and low risk of resistance. For individuals with HIV, treatment initiation is recommended immediately upon diagnosis, regardless of the CD4 count or viral load, as early ART has been shown to significantly reduce morbidity, mortality, and the risk of transmission. In these settings, comprehensive care models are often in place, which include counseling, adherence support, and regular monitoring for side effects and comorbidities^[44]^.

In contrast, low- and middle-income countries, including many in sub-Saharan Africa where the burden of HIV is highest, face more significant challenges in implementing the test and treat strategy. Despite these challenges, ART has become more accessible due to global initiatives, such as the U.S. President’s Emergency Plan for AIDS Relief (PEPFAR) and the Global Fund. In these settings, first-line ART typically consists of a combination of tenofovir, lamivudine (or emtricitabine), and efavirenz (an NNRTI), although, in recent years, there has been a shift toward using INSTIs, like dolutegravir, in first-line therapy due to its improved efficacy and fewer side effects. The World Health Organization (WHO) has recommended dolutegravir as the preferred first-line regimen for HIV treatment, particularly in resource-limited settings. Challenges in these regions include limited access to diagnostic tools, trained healthcare workers, and ongoing issues related to stigma and discrimination. Despite these barriers, the test and treat approach has shown promise, with efforts focused on improving access to care, enhancing testing capabilities, and increasing public awareness^[45]^. The test and treat approach is also gaining traction in middle-income countries, where healthcare systems are evolving rapidly, but access to ART is still limited in some areas. Treatment regimens in these countries often mirror those in high-income nations, but there may be differences in the availability of newer drugs and diagnostic tools. In addition, countries in this group face challenges related to socioeconomic disparities and the growing burden of non-communicable diseases, which complicate the management of HIV^[51^-^58]^. Some countries have adopted policies that prioritize high-risk populations, such as key populations (men who have sex with men, sex workers, and people who inject drugs) or individuals with comorbidities, for immediate treatment initiation. However, the success of the test and treat approach in middle-income countries hinges on scaling up healthcare infrastructure, improving access to medications, and addressing social determinants of health, including education, stigma, and health insurance coverage^[46]^.

## The current state of available and common ART treatment choices and costs in Africa

The state of available antiretroviral therapy (ART) in Africa has evolved significantly over the past few decades, driven by both national and international efforts to improve access to HIV treatment across the continent. Africa remains the region with the highest burden of HIV globally, accounting for over two-thirds of the world’s HIV-positive population. Despite substantial progress in the availability of ART, challenges related to cost, access, healthcare infrastructure, and adherence continue to hinder optimal HIV care in many parts of the continent. ART regimens in Africa generally follow the World Health Organization (WHO) guidelines, which emphasize early treatment initiation to achieve viral suppression and prevent transmission. However, the choice of ART regimens and their availability vary from country to country, influenced by national healthcare policies, local pharmaceutical industries, and international support mechanisms like PEPFAR and the Global Fund^[47,48]^. In many African countries, first-line ART typically consists of a combination of three drugs: two nucleoside reverse transcriptase inhibitors (NRTIs) and one non-nucleoside reverse transcriptase inhibitor (NNRTI). A common regimen includes tenofovir (TDF), lamivudine (3TC), and efavirenz (EFV), a combination that has been widely used due to its proven efficacy and relatively low cost. However, as resistance to NNRTIs increases, there has been a shift toward integrating integrase strand transfer inhibitors (INSTIs) into first-line therapy. Dolutegravir (DTG), an INSTI, has emerged as the preferred first-line treatment in many African countries due to its potent antiviral effects, fewer side effects, and lower risk of developing resistance. WHO included dolutegravir as part of its updated ART guidelines for resource-limited settings, further bolstering its use across the continent. The shift toward INSTIs is a significant improvement in the ART landscape, as dolutegravir and other newer drugs have shown superior efficacy in maintaining viral suppression and improving treatment outcomes^[49,50]^.

The cost of ART remains a significant barrier in many African countries, despite a global reduction in the price of antiretroviral drugs over the past two decades. While the price of first-line ART regimens like TDF/3TC/EFV has dropped substantially, the newer, more effective regimens such as dolutegravir-based therapies still carry a higher cost, though their prices have also been decreasing due to competition and generic production. For instance, the cost of generic dolutegravir can be as low as $50 per person annually, which is a significant improvement compared to the previous first-line regimens, which cost around $150 per person annually. This reduction has made it more feasible for African countries to adopt newer therapies, but in many settings, the affordability of ART remains a challenge, particularly for countries with limited healthcare budgets. The role of international donors, such as PEPFAR, the Global Fund, and UNAIDS, in funding ART programs has been critical in bridging these gaps. Through such support, millions of people in sub-Saharan Africa have gained access to life-saving medications, although sustainability remains a concern^[51,52]^. Healthcare infrastructure, including the availability of skilled healthcare workers and diagnostic tools, also plays a pivotal role in the accessibility and effectiveness of ART in Africa. Many rural areas, where the HIV burden is often highest, continue to face shortages in healthcare personnel and medical facilities^[59^-^62]^. Long-travel distances to clinics, coupled with insufficient resources for patient monitoring and adherence support, often affect treatment outcomes. Moreover, while ART is widely available in urban areas, reaching remote and underserved populations remains a key challenge. Efforts to decentralize HIV treatment services, expand mobile health interventions, and involve community health workers have shown promise in improving access to ART, particularly in rural and hard-to-reach regions. Additionally, the introduction of single-pill combinations (fixed-dose combinations) has simplified treatment regimens and improved adherence, as patients find it easier to take one pill per day instead of several^60^. Furthermore, the increasing availability of HIV self-testing and point-of-care diagnostics has the potential to transform the ART landscape in Africa by enabling earlier diagnosis and treatment initiation, even in the most resource-limited settings. However, the full benefits of these advancements are contingent upon continued improvements in healthcare infrastructure, education, and awareness, as well as addressing social and structural barriers, such as stigma and discrimination that hinder access to care^[53]^.

## Supporting health system strengthening

PPPs contribute significantly to strengthening health systems, making them more resilient and capable of delivering comprehensive HIV services^[48]^. These partnerships often include investments in healthcare infrastructure, capacity building, and the training of healthcare workers. In Zambia, a partnership between the Ministry of Health and several international companies has resulted in the construction and renovation of health facilities, as well as extensive training programs for healthcare providers on the latest HIV treatment and prevention protocols. Additionally, PPPs have facilitated the deployment of mobile health units and telemedicine services, bringing healthcare closer to remote and underserved populations. In Tanzania, collaboration between a mobile telecommunications company and a health NGO has enabled the establishment of mobile clinics that provide HIV testing, counseling, and treatment services in rural areas, significantly improving access to care.

## Innovations in HIV research and development

The private sector’s involvement in HIV research and development has led to significant advancements in HIV prevention and treatment^[49]^. Collaborations between pharmaceutical companies, research institutions, and governments have accelerated the development of new medications, vaccines, and diagnostic tools. For example, a partnership between an international pharmaceutical company and African research institutes has been pivotal in advancing HIV vaccine trials, bringing the world closer to finding an effective vaccine. Moreover, these partnerships often involve the transfer of knowledge and technology, enhancing local research capabilities and fostering innovation within African countries. In South Africa, a PPP focused on HIV research has established state-of-the-art laboratories and training programs for local scientists, contributing to the country’s capacity to conduct cutting-edge research and develop new solutions to combat HIV.

## Economic empowerment and social support programs

PPPs have recognized the importance of addressing the social and economic determinants of health in the fight against HIV^[38]^. Many partnerships include programs aimed at economic empowerment and social support, which are critical for the long-term success of HIV prevention and treatment efforts. For instance, in Uganda, a partnership between an international NGO and a global corporation has launched initiatives that provide vocational training and microfinance opportunities for people living with HIV, helping them achieve financial independence and stability. These programs often include education and awareness campaigns that challenge stigma and discrimination, fostering more supportive and inclusive communities. In Nigeria, collaboration between the government and a multinational company has funded extensive public education campaigns that promote understanding and acceptance of people living with HIV, reducing stigma and encouraging more individuals to seek testing and treatment.

## Challenges

1. **Stigma and Discrimination:** Stigma and discrimination against people living with HIV remain pervasive across many African communities. These social barriers discourage individuals from seeking testing, treatment, and support services, perpetuating the cycle of transmission and late diagnosis. Efforts to combat stigma through education and awareness campaigns must continue to be a priority^[50^–^60]^.
2. **Healthcare Infrastructure:** In many regions, inadequate healthcare infrastructure limits the capacity to deliver comprehensive HIV services^[53]^. This includes shortages of healthcare facilities, medical supplies, and trained personnel. Rural and remote areas are particularly affected, where healthcare access is often limited. Strengthening health systems and improving infrastructure are critical to addressing these gaps.
3. **Funding and sustainability:** While international funding has played a crucial role in HIV response, reliance on external financial support poses a risk to the sustainability of programs^[54]^. Economic fluctuations and changing donor priorities can lead to funding shortfalls. Developing sustainable financing models and increasing domestic investment in HIV programs are essential for long-term success.
4. **Political Instability:** Political instability and conflicts in some African countries disrupt healthcare services, making it difficult to implement and sustain HIV prevention and treatment initiatives. Stable governance and political commitment are vital for the continuity and effectiveness of HIV programs.
5. **Integration with Other Health Services:** Although integrating HIV services with other health services has shown benefits, challenges remain in achieving seamless integration. Coordination between different health programs and sectors can be complex, requiring effective communication and collaboration. Ensuring that HIV services are fully integrated into primary healthcare systems is essential for comprehensive care.
6. **Access to Innovations:** Despite advancements in HIV treatment and prevention technologies, access to these innovations remains uneven. High costs and logistical challenges often limit the availability of new medications and diagnostic tools in resource-limited settings. Ensuring equitable access to the latest HIV technologies is crucial for improving health outcomes.

## Future directions

1. **Community-Led Approaches:** Empowering communities to take a leading role in HIV prevention and treatment can enhance the effectiveness and sustainability of interventions^[55]^. Community-led monitoring, peer education, and support programs can build trust, reduce stigma, and ensure that services are tailored to local needs. Strengthening the capacity of community-based organizations and involving them in decision-making processes are key steps forward.
2. **Innovative Financing Models:** Exploring new financing mechanisms, such as social impact bonds, blended finance, and public-private partnerships, can help secure sustainable funding for HIV programs. Engaging the private sector and leveraging philanthropic contributions can also provide additional resources. Creating financial resilience within HIV programs will ensure their continuity amid changing economic landscapes^[55]^.
3. **Digital Health Solutions:** Leveraging digital technologies can enhance the reach and efficiency of HIV services. Mobile health (mHealth) applications, telemedicine, and electronic health records can improve patient monitoring, adherence to treatment, and access to information. Expanding the use of digital health tools, particularly in remote and underserved areas, can bridge gaps in healthcare delivery.
4. **Focus on Key Populations:** Tailoring HIV interventions to the needs of key populations, such as sex workers, men who have sex with men, and people who inject drugs, is crucial for addressing the epidemic. These groups often face higher risks and barriers to accessing services. Developing targeted programs and policies that protect their rights and provide comprehensive care is essential^[54]^.
5. **Strengthening Political Commitment:** Ensuring sustained political commitment to HIV/AIDS response is vital for long-term success. Governments must prioritize HIV in their national health agendas, allocate adequate resources, and implement policies that support comprehensive care. Advocacy efforts should continue to focus on securing political will and accountability.
6. **Research and Innovation:** Continued investment in research and development is necessary to discover new prevention and treatment options, such as vaccines and long-acting therapies. Collaborations between African research institutions, international organizations, and the private sector can accelerate progress. Promoting innovation and ensuring that new developments are accessible to those in need will be critical.
7. **Addressing Social Determinants of Health:** HIV prevention and treatment efforts must address the broader social determinants of health, such as poverty, gender inequality, and education. Integrating HIV services with programs that promote economic empowerment, education, and gender equity can create a more supportive environment for individuals to protect their health and well-being.
8. **Enhanced Monitoring and Evaluation:** Robust monitoring and evaluation systems are essential for assessing the effectiveness of HIV programs and making data-driven decisions. Investing in data collection, analysis, and reporting can help identify gaps, measure progress, and adapt strategies to changing circumstances. Transparency and accountability in reporting outcomes will also build trust among stakeholders.

## Conclusion

Collaborative strategies have proven to be essential in the ongoing fight against HIV in Africa, where the epidemic continues to have a disproportionate impact on public health. Through multi-sectoral approaches, community-based interventions, and international partnerships, significant progress has been made in reducing HIV transmission and improving access to prevention, testing, and treatment services. Key to this success has been the involvement of local communities, healthcare systems, and governments, as well as regional and global collaborations that have provided vital resources and technical support.

## Data Availability

Not applicable.
